# *Cannabis* Systematics at the Levels of Family, Genus, and Species

**DOI:** 10.1089/can.2018.0039

**Published:** 2018-10-01

**Authors:** John M. McPartland

**Affiliations:** ^1^ Department of Molecular Biology, GW Pharmaceuticals, Cambridge, United Kingdom.; ^2^ Department of Family Medicine, University of Vermont, Burlington, Vermont.

**Keywords:** Cannabaceae, *Cannabis sativa*, center of origin, barcode, molecular clock, palynology

## Abstract

New concepts are reviewed in *Cannabis* systematics, including phylogenetics and nomenclature. The family *Cannabaceae* now includes *Cannabis*, *Humulus*, and eight genera formerly in the *Celtidaceae*. Grouping *Cannabis*, *Humulus*, and *Celtis* actually goes back 250 years. Print fossil of the extinct genus *Dorofeevia* (=*Humularia*) reveals that *Cannabis* lost a sibling perhaps 20 million years ago (mya). *Cannabis* print fossils are rare (*n*=3 worldwide), making it difficult to determine when and where she evolved. A molecular clock analysis with chloroplast DNA (cpDNA) suggests *Cannabis* and *Humulus* diverged 27.8 mya. Microfossil (fossil pollen) data point to a center of origin in the northeastern Tibetan Plateau. Fossil pollen indicates that *Cannabis* dispersed to Europe by 1.8–1.2 mya. Mapping pollen distribution over time suggests that European *Cannabis* went through repeated genetic bottlenecks, when the population shrank during range contractions. Genetic drift in this population likely initiated allopatric differences between European *Cannabis sativa* (cannabidiol [CBD]>Δ^9^-tetrahydrocannabinol [THC]) and Asian *Cannabis indica* (THC>CBD). DNA barcode analysis supports the separation of these taxa at a subspecies level, and recognizing the formal nomenclature of *C. sativa* subsp. *sativa* and *C. sativa* subsp. *indica*. Herbarium specimens reveal that field botanists during the 18th–20th centuries applied these names to their collections rather capriciously. This may have skewed taxonomic determinations by Vavilov and Schultes, ultimately giving rise to today's vernacular taxonomy of “Sativa” and “Indica,” which totally misaligns with formal *C. sativa* and *C. indica*. Ubiquitous interbreeding and hybridization of “Sativa” and “Indica” has rendered their distinctions almost meaningless.

## Introduction

Taxonomy includes classification (the identification and categorization of organisms) and nomenclature (the naming and describing of organisms). Taxonomy, in the light of evolution, becomes systematics: the evolutional relationships among living things. Classification, in the light of evolution, becomes phylogenetics: the genealogical study of relationships among individuals and groups in a nested hierarchy.

This review of *Cannabis* systematics will consist of four sections: (1) the family *Cannabaceae* with the recent addition of former *Celtidaceae*; (2) the genus *Cannabis*, and when and where she evolved; (3) the species *Cannabis sativa*, including two subspecies: *C. sativa* subsp. *sativa* and *C. sativa* subsp. *indica*; (4). the vernacular taxonomy of “Sativa” and “Indica.”

## The Family *Cannabaceae*

The family *Cannabaceae* currently consists of *Cannabis* and *Humulus*, plus eight genera formerly in the *Celtidaceae: Celtis*, *Pteroceltis*, *Aphananthe*, *Chaetachme*, *Gironniera*, *Lozanella*, *Trema*, and *Parasponia*.^1^ Some botanists combine *Parasponia* and *Trema*, but *Parasponia* species uniquely form nitrogen-fixing nodules in symbiosis with rhizobial bacteria. This trait is shared only by legumes. In contrast, other *Cannabaceae* form a symbiosis with arbuscular mycorrhizal fungi, including *Cannabis*.^2^ Family *Cannabaceae* now includes about 170 species.

Cesalpino^3^ first elucidated taxonomic affinities between *Cannabis* and her sister genus *Humulus* in 1583. Before him, botanists classified *Cannabis* with phylogenetically unrelated plants based on leaf shape, human usage, and other totally artificial characters. Cesalpino was an Aristotelian essentialist; he reasoned that plants should be classified by the morphology of their most essential functions—reproduction (flowers and fruits), and nutrition (xylem and phloem).

Schultes^4^ summarizes, “the earliest trend in taxonomic works was to include *Cannabis* in the *Urticaceae*; that in the last half of the last century [19^th^] and the early part of this century [20^th^], most authorities favoured the *Moraceae*; that the modern tendency appears to maintain the family *Cannabaceae* as separate from these.” Schultes is widely quoted or paraphrased, but he presented a simplified history of taxonomy.

Early taxonomists lumped together members of the *Urticaceae*, *Moraceae,* and *Cannabaceae*, and referred to these amalgamated entities by a variety of names. For example, Adanson^5^ lumped 11 genera in 1763: *Cannabis*, *Humulus*, *Celtis*, two *Urticaceae* genera, four *Moraceae* genera, and two unrelated genera. His accuracy (percentage of genera now placed in *Cannabaceae*, *Urticaceae*, or *Moraceae*) was 9 out of 11, or 82%. Some other early concepts are presented in Table 1.

**Table 1.** **Some Early Plant Families into Which *Cannabis* and *Humulus* Have Been Classified, Listed Chronologically**

Author (date)	Family name (and subfamily limited to *Cannabis* and *Humulus* if designated)	Number of genera in family (and subfamily where designated) now classified in *Cannabaceae-Urticaceae-Moraceae-Celtidaceae-*other, with percentage accuracy
Adanson (1763)^5^	*Castaneaceae* Section III	2-2-4-1-2, 82%
Linnaeus (1764)^6^	*Scabridae*	2-3-3-1-4, 69%
Lamarck (1788)^7^	*Figuiers* (*Moraceae*) Section II	2-4-0-0-1, 86%
de Jussieu (1789)^8^	*Urticae* Section II	2-7-2-0-2, 85%
Batsch (1802)^9^	*Scabridae*	2-7-5-0-5, 74%
	(section *Exalbuminosa*)	(2-0-0-0-0, 100%)
Martynov (1820)^10^	*Cannabaceae*	2-0-0-0-0, 100%
Blume (1825)^11^	*Urticeae*	1-3-7-1-5, 71%
	(section *Cannabineae*)	(1-0-0-0-0, 100%)
Gaudichaud-Beaupré (1826)^12^	*Urticeae*	2-26-10-1-9, 81%
	(section *Cannabineae*)	(2-0-0-0-0, 100%)
Nees von Esenbeck et al. (1835)^13^	*Urticaceae*	2-2-2-1-1, 88%
	(tribe *Cannabinae*)	(2-0-0-0-0, 100%)
Lindley (1836)^14^	*Urticaceae*	2-27-18-0-15, 76%
	(subfam. *Cannabineae*)	(2-0-0-0-0, 100%)
Endlicher (1837)^15^	*Cannabineae*	2-0-0-0-0, 100%
Lindley (1846)^16^	*Cannabinaceae*	2-0-0-0-0, 100%
Bentham and Hooker (1880)^17^	*Urticaceae*	2-44-48-8-8, 97%
	(tribe *Cannabineae*)	(2-0-0-0-0, 100%)
Engler and Prantl (1889)^18^	*Moraceae*	2-6-46-0-0, 100%
	(subfam. *Cannaboideae*)	(2-0-0-0-0, 100%)
Cronquist (1968)^19^	*Cannabaceae*	2-0-0-0-0, 100%
Angiosperm Phylogeny Group (2003)^1^	*Cannabaceae*	2-0-0-0-8, 100%

Adanson^5^ was more accurate than Linnaeus,^6^ but Lamarck^7^ outdid them both—although only Adanson accurately combined *Cannabis*, *Humulus*, and *Celtis*. Lamarck^7^ first placed *Cannabis* and *Humulus* in *Moraceae* (although using French instead of Latin, as *Figuiers*), and de Jussieu^8^ first placed *Cannabis* and *Humulus* in *Urticaceae* (spelling it *Urticae*).

Several 18th century taxonomists followed Adanson and grouped *Cannabis*, *Humulus*, and *Celtis*,^11–13^ but Adanson's concept lost recognition thereafter. Batsch^9^ first segregated *Cannabis* and *Humulus* into their own subfamily. Martynov^10^ elevated the pair to a family rank, and coined the name *Cannabaceae*. Subsequent botanists, unaware of Martynov, coined *Cannabineae*^15^ and *Cannabinaceae*.^16^ These incorrect spellings still appear in the literature. Others continued to place *Cannabis* into the *Urticaceae* or *Moraceae*.^17,18^ Not until the mid-20th century has *Cannabaceae* come into common usage.^19^

In 2003, Angiosperm Phylogeny Group^1^ merged *Cannabaceae* with *Celtidaceae* based on genetic (DNA) evidence. The merger is morphologically counterintuitive—*Cannabis* and *Humulus* species are herbal plants, whereas the *Celtidaceae* are woody trees. Yang et al.^20^ provide the latest analysis, utilizing four chloroplast DNA (cpDNA) genes (*trnL-trnF*, *rbcL*, *atpB-rbcL*, and *rps16*). Their results strongly support this expanded family as a monophyletic group. *Cannabis* and *Humulus* form a clade that nests within former *Celtidaceae* genera. Naming the family *Celtidaceae* might better reflect its members, but the name *Cannabaceae* is older and therefore holds nomenclatural priority.

Genetic (DNA) data should be congruent with phenotypic characters (morphology, phytochemistry, host–parasite relationships, etc.). Congruency can be tested with a procedure called character mapping, also known as ancestral reconstruction—assessing phenotypic evolution by laying phenotypic characters upon a phylogenetic tree. Yang et al.^20^ reconstructed eight ancestral characters in the *Cannabaceae.*

*Cannabis* and *Humulus* shared only three of the eight ancestral characters: triporate pollen grains, imbricate flower aestivation, and a persistent perianth (domesticated *Cannabis* has a deciduous perianth). Ancestral stipule arrangement—extrapetiolar stipules—was expressed by *Cannabis*, with a shift to interpetiolar stipules in *Humulus*. An ancestral seed coat character—a seed coat lacking microscopic holes—was expressed by *Cannabis*, with a shift to a seed coat with holes in *Humulus*. Three other *Cannabaceae* ancestral characters were not shared by *Cannabis* and *Humulus*: monoecy (*Cannabis* and *Humulus* are dioecious), alternate leaf arrangement (*Cannabis* and *Humulus* have both alternate and opposite leaves), and fleshy drupes (*Cannabis* and *Humulus* have achenes).

In their younger days, *Cannabis* and *Humulus* lost a sibling genus. Dorofeev^21^ described and illustrated fruits of two species in an extinct genus: *Humularia reticulata* and *Humulus tymensis*. Dorofeev found both species in central Siberia, and dated them to the Oligocene Epoch, 33.9–23.03 million years ago (mya). Collinson^22^ re-examined Dorofeev's fossils, and confirmed that they represent an extinct taxon within *Cannabaceae.* Grudzinskaya^23^ assigned a new (legitimate) name, *Dorofeevia*, to the extinct genus.

## The Genus *Cannabis*

**FIG. 1.**
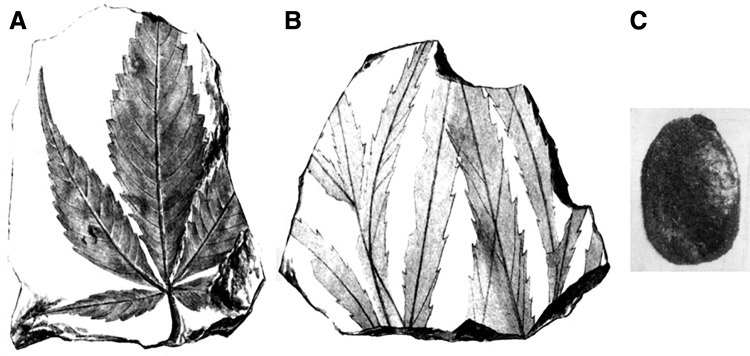
Macrofossils identified as *Cannabis* (not to scale). **(A, B)** Are from Friedrich,^28^ and **(C)** Is from Palamarev.^25^ **(A, B)** Reproduced from a publication whose copyright has expired; **(C)** Reproduced courtesy of Vladimir Bozukov, Bulgarian Academy of Sciences.

Accurately determining when *Cannabis* evolved is difficult, because the genus lacks a robust print fossil record (impressions of leaves or fruits in rocks). Friedrich^24^ found fossil leaves in Germany that he named *Cannabis oligocaenica* (Fig. 1A, B). Friedrich did not date his fossil, but his species epithet refers to the Oligocene Epoch. Palamarev^25^ identified a fossil seed (achene) as “*Cannabis* sp.” in Bulgaria (Fig. 1C). He dated the fossil to the “Pontian age,” 7.3–5.3 mya, which is the end of the Miocene Epoch (23.03–5.33 mya).

Dorofeev^26^ described and illustrated a fossil seed, “*Cannabis* sp.,” from the Miocene Epoch, found in Siberia. He had previously assigned the fossil to *Humulus lupulus*.^27^ Dorofeev^21^ changed his mind again, and reidentified the fossil as an extinct species, *Humulus irtyshensis*. He described three other extinct *Humulus* species, all from Siberia: *H. strumulosus*, dating to the Oligocene,^21^ *H. minimus*, also from the Oligocene,^21^ and *H. rotundatus*, dating to the Miocene.^27^

MacGinitie^29^ found leaf prints in Florissant, Colorado, that he named *Vitus florissantella*. He subsequently renamed one of the fossils *Humulus florissantella*.^30^ The fossil lacks diagnostic fruits; assigning the leaf to either *Vitus* or *Humulus* is debatable. The Florissant fossil bed has been dated to 34.07 mya using ^40^Ar/^39^Ar radiometric dating.^31^

For organisms like *Cannabis* that lack a good fossil record, a “molecular clock” can estimate when they diverged from other organisms. The molecular clock uses DNA to measure time, because DNA accumulates random mutations at a fairly constant rate. Some species might evolve at different rates, however, so computer algorithms now allow for variable rates between lineages in a phylogenetic tree, and calibrate the clock with fossil dates of related plants.

McPartland and Guy^32^ used a variable rate-smoothing algorithm, calibrated with four fossil records. They constructed a phylogenetic tree using cpDNA sequences (*rbcL*+*trnL-trnF* intergenic spacer data obtained from Gilmore,^33^ and other sequences from Genbank). ClustalX (version 2.0) was used to build a multiple sequence alignment. A maximum likelihood phylogenetic tree was constructed with PAUP* (version 4.0b10), using Modeltest 3.06 to select an optimal ML model. Divergence dates were estimated with r8s (version 1.70, nonparametric algorithm).

**FIG. 2.**
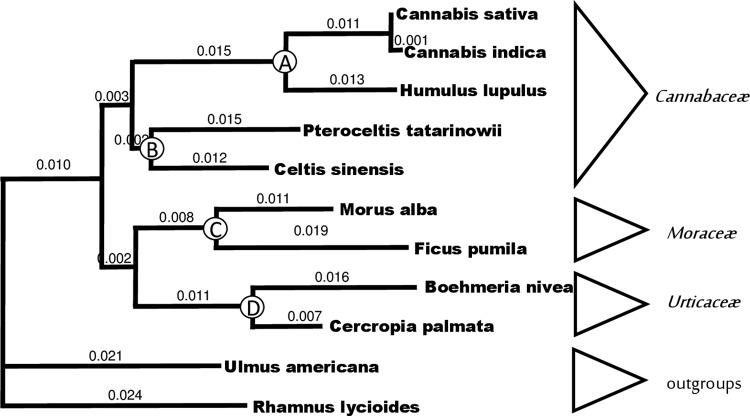
Phylogenetic tree used for calculating divergence dates.

The clock was calibrated with fossils of *Humulus* (node A, 28–16 mya^34^), *Celtis* (node B, 65–56 mya^35^), *Morus* and *Ficus* (node C, 56–34 mya^22^), and *Boehmeria* (node D, 60–34 mya^22,34^). The phylogenetic tree with branch lengths and calibration nodes appears in Figure 2. They estimated that *Humulus* and *Cannabis* diverged from a common ancestor 27.8 mya. *C. sativa* and *Cannabis indica* diverged 1.05 million years ago, but this was not published because the taxa differ at only one nucleotide site.

Every species occupies an indigenous geographical area, its native range. An Iraqi agronomist named Ibn Wahshīyah first speculated upon the native range of *C. sativa* in 904 AD. He proposed that *šāhdānaj* (*C. sativa*) came from India or perhaps China.^36^ Starting with Linnaeus, the native range of a cultivated plant has been deduced by locating its congeners, or wild relatives. Finding wild relatives is complicated by the fact that *C. sativa* easily escapes cultivation, and reacquires wild-type characteristics (*a.k.a.*, it becomes naturalized, and survives as a “feral escape”). *C. sativa* reacquires wild-type characteristics in as little as 50 generations (years).^37^

Linnaeus^38^ knew *C. sativa* as a cultivated plant in Europe, so he deduced its native range was elsewhere: *India Orientali* (encompassing the Indian subcontinent, southeastern Asia, and the Malay Archipelago), *Japonia* (Japan), and *Malabaria* (the Malabar coast of southwest India). Some botanists considered *C. sativa* native to Europe, rather than Asia.^39,40^ Winterschmidt^41^ recognized two species, *C. sativa* and *Cannabis chinensis*, with their native ranges in *Russland* and *Ostindien*, respectively. Lamarck^42^ recognized two species, *C. sativa* and *C. indica*. He suggested that *C. sativa* grew *croît naturellement* in Persia and *presque naturalisée* in Europe, whereas *C. indica* originated in India.

Within a species's native range lies its center of origin, from whence it dispersed. De Candolle^43^ offered Central Asia as the *C. sativa* center of origin. “*The species has been found wild, beyond a doubt, to the south of the Caspian Sea* (Azerbaijan, Iran, Turkmenistan), *near the Irtysch* (Irtysh River, arising in the Altai, flowing through Kazakhstan to western Siberia), *in the desert of Kirghiz* (the Kazakh steppe), *beyond Lake Baikal in Dahuria* (Transbaikal). *The antiquity of the cultivation of hemp in China leads me to believe that its area extended further to the east…*” He added that *C. sativa* grew “*almost wild in Persia*,” but he doubted it was indigenous there, “*since in that case the Greeks and the Hebrews would have known of it at an earlier period.*” *C. sativa* was not native to the Levant, because the ancient Egyptians and Hebrews did not know hemp.

Due to the paucity of *Cannabis* print fossils, paleobotanists have turned to microfossils, in the form of fossil pollen or subfossil (noncarbonized) pollen. Hundreds of fossil pollen studies (FPSs) have reported *Cannabis* or *Humulus* pollen. Many paleobotanists, confronted with the morphological similarities between *Cannabis* and *Humulus* pollen grains, resort to collective names, for example, *Cannabis/Humulus* or *Cannabaceae*. These aggregate data can be dissected by using ecological proxies, instead of grain morphology. *Cannabis/Humulus* pollen in a steppe assemblage (occurring with *Poaceae*, *Artemisia*, and *Chenopodiaceae* pollen) is consistent with wild-type *Cannabis*. *Cannabis/Humulus* pollen in a mesophytic forest assemblage (occurring with *Alnus*, *Salix*, and *Populus* pollen) is consistent with *Humulus*.^44^

A meta-analysis of 88 FPSs in Asia used these methods.^45^ The oldest pollen in a steppe assemblage was located in Níngxià Province, China, and dated to 19.6 mya. A map of the FPSs constructed with geographical information system (GIS) software identified the northeastern Tibetan Plateau as the *Cannabis* center of origin. This geographical region, during the Oligocene, agrees with two hypotheses regarding the evolution of cannabinoid biosynthesis: (1) Cannabinoids protect plants from ultraviolet light (UV_B_) at higher altitudes, generated by the Tibetan uplift. (2) Cannabinoids deter vertebrate herbivores—the expansion of steppe during the Oligocene led to the evolution of Central Asian animals that feed on *Cannabis* today, such as Ungulates (horses), Rodentia (some families of rats, mice, and hamsters), Lagomorpha (rabbits and pikas), and Columbiformes (pigeons and doves).

A meta-analysis of 479 FPSs in Europe^44^ addressed the generally accepted concept that the dispersal of *C. sativa* from Asia to Europe depended upon human transport. The Scythians are often implicated, *ca.* 700 BCE.^43^ The meta-analysis identified *Cannabis* pollen in Europe that predated the Scythians, and even predated *Homo sapiens*. The oldest pollen dated to the Eopleistocene (1.8–1.2 mya), and came from the Upper Volga River basin in Russia. A prehistoric dispersal to Europe should not come as a surprise, because three other *Cannabaceae* genera spread through Eurasia, even to North America: *Celtis*,^46^ *Humulus*,^46^ and *Pteroceltis*.^47^

During the Last Glacial Maximum, northern Europe was covered by ice, with a southern margin between 52° N (midland England) and 56° (north of Moscow). Tree species retreated to glacial refugia in southern Europe, south of around 45° N. Between these latitudes, GIS mapping revealed a belt of *Cannabis* pollen spanning Europe.^44^

Following the warm and wet Holocene Climactic Optimum (7–6 kya), forests replaced steppe, and *Cannabis* retreated to steppe refugia in the Pontic steppe and the Mediterranean coast.^44^ This pattern was repeated through several cycles of stadials (ice ages) and interstadials (warmer, wetter periods). *Cannabis* went through repeated “genetic bottlenecks,” when the population shrank to small numbers during range contractions. Small populations experience genetic drift, where new genotypes arise randomly. Conventional wisdom states that differences between *C. sativa* and *C. indica* are due to human selection, and therefore not “natural.” Instead, FPSs suggests that genetic drift initiated allopatric differences between European *C. sativa* and Asian *C. indica*.

## The Species *C. sativa*

The publication of Linnaeus's *Species Plantarum* in 1753^48^ is treated as the starting point of botanical nomenclature, hence *C. sativa* has nomenclatural priority. Thirty-two years later, Lamarck^42^ coined *C. indica* for plants with provenance from India, Southeast Asia, and South Africa. Lamarck's description of *C. indica* differed from his description of *C. sativa* by eight “very distinct” morphological characters in stalks, branching habitus, leaflets, and flowers. Lamarck also described chemotaxonomic differences: *C. indica* produced a strong odor, and was psychoactive, “The principal effect of this plant consists of going to the head, disrupting the brain, where it produces a sort of drunkenness that makes one forget one's sorrows, and produces a strong gaiety.”

**FIG. 3.**
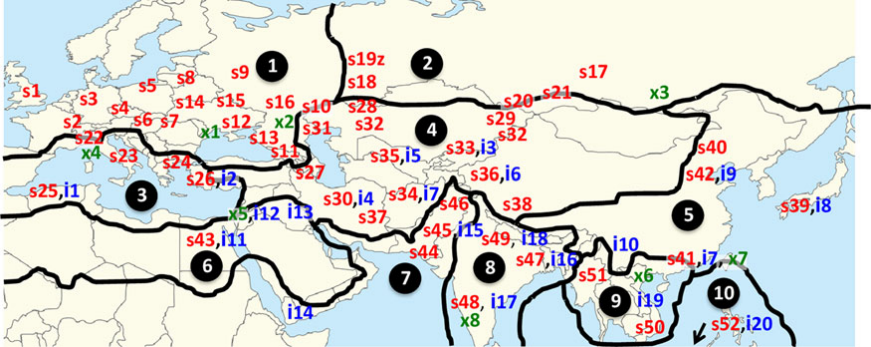
Taxon names applied by field botanists. Locations of “*C. sativa*” labeled alphanumerically, s1, s2, and so on. Locations of “*C. indica*” labeled alphanumerically, i1, i2, and so on. Locations of other *Cannabis* taxa labeled alphanumerically, x1, x2, and so on. Base map shows the boundaries of 10 floristic regions (Takhtajan 1986). Map adapted from McPartland and Guy,^51^ which links alphanumerical sites with literature citations.

Linnaeus's disciples soon rendered subjective decisions regarding Lamarck's concept of a polyspecific genus. Persoon^49^ reduced Lamarck's species to *C. sativa β indica*. Persoon used Greek letters to indicate a varietal rank.^50^ There is evidence of cultural bias influencing these taxonomic decisions, arising from personality cults surrounding Linnaeus and Lamarck.^51^ It is expected that the distributions of *C. sativa* and *C. indica* (at species or subspecies rank) should occupy separate floristic regions. The distribution of plants that field botanists identified as *C. sativa* or *C. indica* in the 18th–19th centuries is presented in Figure 3.

The map shows no hint of endemic distribution in specific floristic regions, for either *C. sativa* or *C. indica*. Field botanists assigned names according to their cultural biases. In general, Linnaeus's disciples from Scandinavia and Great Britain used “*C. sativa*.” Lamarck's disciples from France and Francophone Russia used “*C. indica*” or coined new taxa, such as *C. chinensis* and *Cannabis gigantea*. Reports of “*C. sativa*” in the Indian floristic region (region 8 in Fig. 3) are particularly striking. Examining field botanists' collections in 15 worldwide herbaria verified erroneous determinations.^51^

*C. sativa* and *C. indica* can be separated by morphology (*C. sativa* is taller with a fibrous stalk, whereas *C. indica* is shorter with a woody stalk), by phytochemistry (*C. sativa* has a cannabinoid ratio of Δ^9^-tetrahydrocannabinol (THC)>cannabidiol [CBD], whereas *C. indica* has a ratio of THC<CBD), and by differences in their original geographic range (*C. sativa* in Europe and *C. indica* in Asia). The taxonomic rank at which we separate *C. sativa* and *C. indica* is the primary issue in *Cannabis* taxonomy: are they different subspecies,^52^ or different species?^53,54^

Taxonomic ranks are relative levels within a hierarchy, and notoriously subjective. Darwin^55^ could not reconcile the continuous process of evolution with the discrete concept of taxonomic ranks. “I was much struck how entirely vague and arbitrary is the distinction between species and varieties.” Generally, different species are reproductively isolated and cannot hybridize, but this is not always true in plants. For example, *Brassica napus* (rapeseed) can hybridize with *Brassica rapa* (a weed); genetically modified *B. napus* has spread transgenic glyphosate resistance to its weedy relative.^56^

“DNA barcodes” make the question of rank less “vague and arbitrary.” The *Consortium for the Barcode of Life* uses the mitochondrial *COI* gene as a DNA barcode to identify animal species. Herbert^57^ proposed a 2.7% difference between two *COI* sequences as the threshold for flagging genetically divergent specimens as distinct species. The low rate of nucleotide substitutions in plant mitochondrial genes precludes the use of *COI* as a plant barcode. Numerous barcodes have been proposed for plants. Kress and Erickson^58^ reported sequence divergences (“barcode gaps”) between plant species: a species threshold of 5.7% for *ITS1*, 2.7% for *trnH-psbA*, 2.1% for *rpoB2*, 1.4% for *rpoC1*, and 1.3% for *rbcL*.

McPartland and Guy^59^ used barcode gaps in five sequences (*rbcL*, *matK*, *trnH-psbA*, *trnL-trnF*, and *ITS1*) to place the *Cannabis* question of rank in context with other plants. Pairwise alignments of sequences were made with BLAST. Differences between aligned sequences were quantified by tallying the number of nucleotide nonidentities as a percentage of the total BLAST alignment. These calculations were repeated with four groups of plants. All sequences were obtained from GenBank (Table 2).

**Table 2.** **Pairwise Barcode Gaps in Five Groups of Plants**

	Taxon comparison	rbcL (%)	matK (%)	trnH-psbA (%)	trnL-trnF (%)	ITS (%)	Mean % ±SEM
1	Apples and oranges	8.6	18.2	19.0	26.6	18.0	18.07±2.869
2	*Solanum lycopericum* and *Solanum tuberosum*	1.00	1.07	5.67	0.40	11.6	3.95±2.134
	*Humulus lupulus* and *Humulus japonicus*	1.03	1.13	3.18	1.0	10.7	3.41±1.869
	*Trema orientalis* and *Trema micrantha*	0.72	0.73	Missing	0.61	8.55	2.65±1.966
	*Fagopyrum esculentum* and *Fagopyrum tataricum*	0.50	2.76	Missing	0.12	7.27	2.66±1.642
	*Panax ginseng* and *Panax pseudoginseng*	1.25	1.22	2.69	1.11	5.57	2.37±0.852
3	*Raphanus sativus* and *Raphanus raphanistrum*	0.0	0.0	0.0	3.54	2.84	1.28±0.789
	*Populus alba* and *Populus trichocarpa*	0.73	0.12	2.95	0.0	2.45	1.25±0.610
	*Oryza sativa* and *Oryza rufipogon*	0.0	0.1	0.89	0.0	3.4	0.879±0.652
	*Gossypium hirsutum* and *Gossypium barbadense*	0.21	0.26	2.09	0.61	0.55	0.743±0.346
4	*Camellia sinensis* var. *sinensis* and *C. sinensis* var*. assamica*	0.0	Missing	0.0	1.22	1.90	0.780±0.471
	*O. sativa* subsp*. indica* and *O. sativa* subsp*. japonica*	0.01	0.0	1.16	0.0	1.30	0.494±0.301
	*Brassica oleracea* var*. capitata* and *B. oleracea* var*. italica*	0.40	Missing	0.0	0.79	0.50	0.406±0.257
	*Acorus calamus* var. *calamus* and *A. calamus* var. *americanus*	0.0	0.0	0.0	0.0	1.52	0.304±0.304
	*Curcumis melo* subsp. *melo* and *C. melo* subsp. *agrestis*	0.0	0.24	Missing	0.63	0.16	0.258±0.134
5	*Cannabis sativa* and *Cannabis indica*	0.08	0	0.40	0.15	1.4	0.406±0.257

Apples and oranges—different genera and different species—express a mean barcode gap of 18.07% over the five sequences. The mean barcode gap in five pairs of related species that cannot hybridize (group 2 in Table 2) is 3.0%±0.3%, which is analogous to the 2.7% threshold for *COI* in animal species. The mean barcode gap in five pairs of closely related species that can hybridize (group 3) is 1.0%±0.1%. The mean barcode gap in five pairs of plants classified at the rank of subspecies or variety (group 4) is 0.43%±0.1%.

For *C. sativa* and *C. indica*, the mean barcode gap is 0.406±0.257. This difference nearly equals the mean of plants at the rank of subspecies or variety. *C. sativa* and *C. indica* should not be considered different species. The proper nomenclature is *C. sativa* subsp. *sativa* and *C. sativa* subsp. *indica*.^52^

Small and Cronquist^52^ erected a formal botanical nomenclature for *C. sativa* that has not been replaced. Their taxonomic concept is relatively simple: a two-step hierarchic classification system. The first step recognizes two subspecies based on THC content in dried female flowering tops, with 0.3% THC as the dividing point. The second step recognizes two varieties within each subspecies, based on their domestication phase:

- *C. sativa* subsp. *sativa* var. *sativa* (low THC, with domestication traits)
- *C. sativa* subsp. *sativa* var. *spontanea* (low THC, wild-type traits)
- *C. sativa* subsp. *indica* var. *indica* (high THC, domestication traits)
- *C. sativa* subsp. *indica* var. *kafiristanica* (high THC, wild-type traits)

The protologs of these four varieties are reviewed elsewhere, including their original descriptions, synonymies, and photographs of the four herbarium type specimens.^51^ That review compared and critiqued other taxonomic models by Vavilov, Schultes, de Meijer, and Hillig. Small's model adheres closest to protolog data (with *C. indica* treated as a subspecies). Importantly, Small and Cronquist^52^ noted that *C. sativa* subsp. *sativa* var. *spontanea* Vavilov 1922^60^ has nomenclatural priority over *C. sativa* var. *ruderalis* Janischevsky 1924.^61^ Regarding the latter taxon, Janischevsky,^61^ also offered an alternative rank at the species level, *C. ruderalis*, but added, “I am inclined to consider it a well marked variety.”

## Vernacular Taxonomy: “Sativa,” “Indica,” and “Ruderalis”

A folk taxonomy of drug-type plants, “Sativa” and “Indica,” has entangled and subsumed the nomenclature of *C. sativa* and *C. indica.* Thousands of websites generalize about the morphological, phytochemical, organoleptic, and clinical properties of these plants. “Sativa” is recommended for treating depression, headaches, nausea, and loss of appetite; it causes a stimulating and energizing type of psychoactivity. “Indica” is recommended for treating insomnia, pain, inflammation, muscle spasms, epilepsy, and glaucoma; it causes a relaxing and sedating psychoactivity.

“Sativa” plants produce more THC than CBD, and a terpenoid profile that smells “herbal” or “sweet.” “Indica” plants produce more CBD than “Sativa,” with a THC-to-CBD ratio closer to 1:1. “Indica” terpenoids impart an acrid or “skunky” aroma. Clarke^62^ first described the unique organoleptic properties of “Indica” plants, as a “slow flat dreary high.”

Small^63^ noted that “Sativa” and “Indica” were “quite inconsistent” with formal nomenclature, because *C. sativa* subsp. *sativa* should strictly apply to nonintoxicant plants. Conflating formal and vernacular taxonomy has begun to muddle otherwise excellent studies that worked with “Sativa” but latinized the taxon as *C. sativa*. This confusion even appeared in the distinguished journal *Nature*.^64^ “Sativa” and “Indica” written in quotation marks mean different things than *C. sativa* and *C. indica* written in italics.

McPartland et al.^65^ derided the inaccuracy of vernacular taxonomy, followed by others.^54,63,66^ Some experts propose jettisoning all vernacular names in favor of a metabolomics classification, “from cultivar to chemovar.”^67,68^ The parade of mistakes leading to “Sativa” and “Indica” is detailed elsewhere.^51^

Briefly, Vavilov^69^ assigned the taxon *C. sativa* to plants cultivated in Afghanistan for hashish. This concept departed from Linnaeus's protolog of *C. sativa* as a fiber-type, nonintoxicant plant from Europe. Vavilov^69^ coined a new taxon, *C. indica* var. *afghanica*, for plants with obovate leaflets, medium height, and profuse branching. Some botanists argue that *afghanica* designates a wild-type plant. But Vavilov's descriptions, illustrations, and other evidence indicate that *afghanica* was a feral escape of cultivated plants.^51,52^

Schultes et al.^70^ assigned the taxon *C. indica* to Afghani plants, and described the taxon having broad, oblanceolate leaflets, densely branched, more or less conical in shape, and very short (< 1.3 m). Designating these plants as *C. indica* was faulty; Lamarck was entirely unfamiliar with Afghani *Cannabis*. Lamarck's protolog of *C. indica* describes plants that are relatively tall, laxly branched, and with narrow leaflets.

**FIG. 4.**
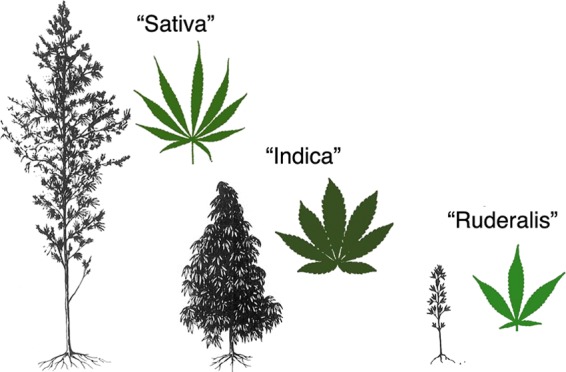
*Cannabis* vernacular taxonomy, image adapted from Anderson,^71^ courtesy of the Harvard University Herbaria and Botany Libraries.

Anderson^71^ repeated the errors by Vavilov and Schultes. He typified *C. indica* with plants that Schultes described in Afghanistan. He assigned *C. sativa* to plants consistent with Lamarck's *C. indica*. Anderson illustrated these concepts in a line drawing (Fig. 4). This illustration has become pervasive on the web as the poster child of vernacular nomenclature.

De Meijer and van Soest^72^ introduced the vernacular taxonomy to peer-reviewed literature: “Indica” refers to plants with broad leaflets, compact habit, and early maturation, typified by plants from Afghanistan. “Sativa” refers to plants with narrow leaflets, slender and tall habit, and late maturation, typified by plants from India and their descendants in Thailand, South and East Africa, Colombia, and Mexico.

Categorizing cannabis as either “Sativa” and “Indica” has become an exercise in futility. Ubiquitous interbreeding and hybridization renders their distinction meaningless. The arbitrariness of these designations is illustrated by “AK-47,” a hybrid that won “Best Sativa” in the 1999 Cannabis Cup, and won “Best Indica” four years later.^73^ More than 30 years ago, unhybridized plants of Indian heritage and Afghani landraces were already difficult to obtain.^62^ Hybridization has largely obliterated population differences, “especially between the two kinds of fiber forms and between the two kinds of marijuana forms.”^74^

Schultes et al.^70^ made another taxonomic error. He eschewed Vavilov's taxon, *C. sativa* var. *spontanea*, in favor of Janischevsky's later synonym, *Cannabis ruderalis*. He then departed from Janischevsky's concept of *C. ruderalis* by applying the taxon to extremely short (≤0.61 m), unbranched plants with broad leaflets from Central Asia, instead of Janischevsky's relatively tall, laxly branched plants with narrow leaflets from southeastern Europe (Janischevsky described plants up to 2.1 m tall). Anderson^71^ illustrated a plant consistent with Schultes, not Janischevsky. One of the first seed bank catalogs illustrated “Ruderalis” plants growing near the Hungary-Ukraine border.^75^ The photos of “Ruderalis” show plants with strong apical dominance and little branching. These traits are consistent with a spontaneous escape of cultivated hemp, and depart from concepts by both Vavilov and Janischevsky.

In today's vernacular taxonomy, “Ruderalis” is applied to plants that exhibit one to three characteristics: CBD≅THC, wild-type morphology, or early flowering (sometimes called “autoflowering,” that is, day-neutral, flowering not induced by light cycle). Some authors have tried to reconcile “Sativa” and “Indica” with formal *C. sativa* and *C. indica*. McPartland et al.^65^ noted that Afghani plants were mislabeled “Indica.” They reassigned “Indica” to Vavilov's taxon, at species rank (*Cannabis afghanica*) or varietal rank (*C. sativa* var. *afghanica*).

In summary, reconciling the vernacular and formal nomenclatures: “Sativa” is really *indica*, “Indica” is actually *afghanica*, and “Ruderalis” is usually *sativa*. All three are varieties of one species, *C. sativa* L.

## Abbreviations Used

CBD: cannabidiol
cpDNA: chloroplast DNA
FPSs: fossil pollen studies
GIS: geographical information system
THC: Δ^9^-tetrahydrocannabinol
